# Basic Life Support Knowledge Among a Nonmedical Population in Jeddah, Saudi Arabia: Cross-Sectional Study

**DOI:** 10.2196/10428

**Published:** 2018-11-28

**Authors:** Ahmed Hussein Subki, Hatan Hisham Mortada, Mohammed Saad Alsallum, Ali Taleb Alattas, Mohammed Ali Almalki, Muhab Mohammed Hindi, Siham Hussein Subki, Wesam Awad Alhejily

**Affiliations:** 1 Department of Internal Medicine Faculty of Medicine King Abdulaziz University Jeddah Saudi Arabia

**Keywords:** basic life support, BLS, cardiopulmonary resuscitation, CPR, awareness, public, knowledge, Jeddah, Saudi Arabia

## Abstract

**Background:**

Providing basic life support (BLS) at the site of an accident is crucial to increase the survival rates of the injured people. It is especially relevant when health care is far away.

**Objective:**

The aim of our study is to assess the BLS knowledge level of the Saudi Arabian population and identify influencing factors associated with level of knowledge about BLS.

**Methods:**

Our study is a cross-sectional descriptive study, which was conducted using a self-administered online questionnaire derived from the BLS practice test. The Saudi population was the target population. The questionnaire was divided into two parts: one contained demographic data and the second part contained questions to test the population’s perception about how to perform BLS techniques properly. The data were collected between July and August 2017. Statistically significant differences were defined as those with a *P* value <.05, and a score of five or more was considered a passing score on the second part. We used SPSS version 21 for data analysis.

**Results:**

Our study included 301 participants. Our participants’ BLS online exam scores ranged from 0 to 10, with a mean of 4.1 (SD 1.7). Only 39.2% (118/301) of the participants passed the test. The percentage of bachelor’s degree or higher holders constituted 60.1% (181/301) of the study population. In addition, higher income was significantly associated with higher scores on the test (*P*=.04).

**Conclusions:**

This study demonstrated that the theoretical knowledge level of BLS among the general population in Jeddah was below average. There is a critical need to increase the public’s exposure to BLS education through raising awareness campaigns and government-funded training programs that aim to curb the incidence of out-of-hospital cardiac arrest mortalities in the Saudi community.

## Introduction

Life-threatening emergencies can occur anytime and anywhere. The lack of training and incompetence to deal with these emergencies can have tragic and legal consequences. Basic resuscitation skills, including prompt and effective cardiopulmonary resuscitation (CPR), increases the survival rate following cardiopulmonary arrest. Theoretical knowledge with practical demonstrations and regular practice with up-to-date recommendations is important in maintaining the capability of basic life support (BLS) and advanced life support (ALS) providers [1]. Saving peoples’ lives involves a sequence of steps that constitute the chain of survival. This includes four stages: early approach to cohesive medical emergency, early initiation of BLS, early defibrillation, and ALS [2].

Sudden cardiac arrest is the most common cause of death worldwide with a large variation in survival rates between different communities [3,4]. Early detection of cardiac arrest and initiation of CPR have been shown to decrease morbidity and mortality [4-6]. A previous study conducted in Arizona showed that a statewide hands-only CPR public awareness campaign increased bystander CPR rates from 28.2% to 39.9% and improved out-of-hospital cardiac arrest (OHCA) survival rates from 3.7% to 9.8% [7]. The number of OHCA cases in the United States is approximately 300,000 and the mortality rate is 92% [8]. The chance of survival increases two-fold if BLS is conducted by the first person to intervene and with the use of automated external defibrillators [8].

Difficulties performing bystander CPR in more developed countries were due to inadequate knowledge or training, absence of skill, lack of confidence, and fear of litigation [9]. In order to provide proper care, the community needs an adequate amount of knowledge and insight on BLS by training either in person or online [10]. An important method to increase CPR and BLS success for any emergency case is to increase public knowledge and understanding of the practical applications of BLS intervention for a successful result after an emergency. An assessment of community knowledge and perceptions of BLS has not been performed in Saudi Arabia.

Our study aims to assess the BLS knowledge level of a Saudi population and to identify demographic factors such as age, gender, nationality, marital status, educational level, and monthly family income related to the level of knowledge about BLS.

## Methods

### Study Design and Data Collection

Our study is a cross-sectional descriptive study that was designed to assess the knowledge and perception about BLS among a Saudi population via an online questionnaire. The data were collected between July and August 2017 in Jeddah, Saudi Arabia.

### Measures

Our questionnaire was developed by BLS provider National Health Care Provider Solutions, who provided permission to use the questionnaire [11]. The questionnaire adhered to the latest American Heart Association and Emergency Cardiovascular Care guidelines [12].

Our questionnaire was an online, multiple-choice, self-administered questionnaire in two parts. The first part focused on the participants’ demographic data, including age, nationality, gender, marital status, education, household income, place of residence, and district. The second part was about the responders’ perceptions about BLS, including the ratio for one rescuer giving CPR, how often should rescuers switch roles when performing two-rescuer CPR, the proper steps for operating an automated external defibrillator, site to check a pulse in a child whose age is between 1 year to puberty, the initial BLS steps in adults, critical characteristics of high-quality CPR, the five steps in the adult chain of survival, the recommended BLS sequence of steps, signs of airway obstruction, and how often the breaths are administered in an adult with an advanced airway in place during two-rescuer CPR (see Multimedia Appendix 1 for the questionnaire). A score of five or more was considered a passing score.

The survey was created initially in English, then translated and back-translated into the Arabic language by two independent translators. Any discrepancy was resolved through consensus.

The inclusion criteria included all males and females in the Saudi population. Participants who refused to participate or had a medical background were excluded.

A link to the questionnaire was distributed to the participants through Facebook and WhatsApp groups. They were informed on the first page of the questionnaire about the study with a disclaimer on it that only those people without a medical background could fill it; if they agreed, they could continue. The definition of “medical background” was left up to the participants to decide, but it included anyone who works or had worked in the health care system or was student in medical, nursing, dental schools, and any health care-related colleges. Basic health knowledge taught in school was not included in our definition. Ethical approval was obtained from the Unit of Biomedical Ethics, Research Committee at King Abdul Aziz University, before data collection started.

### Statistical Analysis

Data were presented as a mean for central tendency with standard deviation for variance. The chi-square test was used to compare different variables. Statistically significant differences were defined as those with a *P*<.05. We used SPSS version 21 for data analysis.

## Results

Our study included 301 responders. Of these, 278 (92.4%) were Saudi Arabian. The response rate was not possible to determine, but this was due to the online nature of the questionnaire. If they did not want to participate they could simply discard the link. The mean age for participants was 24.1 (SD 8.9) years. More than two-thirds of participants were younger than 25 years of age (227/301, 75.4%). The majority of responders were also female (217/301, 72.1%) and single (240/301, 79.7%). Of the respondents, more than half held a bachelor’s degree (174/301, 57.8%) and one-third had a total family income between 5000 SR to 10,000 SR. Most of the participants (245/ 301, 81.4%) lived in Jeddah, Saudi Arabia (see Table 1 for full demographic details of participants. Our participants’ BLS online exam scores ranged from 0 to 10, with a mean of 4.08 (SD 1.71). Only 39.2% (118/301) of participants passed the test. The percentage of participants who passed the exam was compared across various factors (Table 2) using the chi-square test.

**Table 1:** Characteristics of participants (N=301).

Variable			n (%)
**Age^a^**			
	<25 years	225 (75.3)	
	≥25 years	74 (24.7)	
**Nationality**			
	Saudi	278 (92.4)	
	Non-Saudi	23 (7.6)	
**Sex**			
	Male	84 (27.9)	
	Female	217 (72.1)	
**Marital status**			
	Single	240 (79.7)	
	Married	60 (19.9)	
	Divorced	1 (0.3)	
**Educational level**			
	Illiterate	2 (0.7)	
	High school or less	106 (35.2)	
	Professional certificate (noncollege issued)	11 (3.7)	
	Bachelor	174 (57.8)	
	Masters or PhD	8 (2.7)	
**Monthly income (SR)**			
	<5000	34 (11.3)	
	5000 to 10,000	100 (33.2)	
	10,000 to 20,000	98 (32.6)	
	>20,000	69 (22.9)	
**Residence**			
	Jeddah	245 (81.4)	
	Outside Jeddah	56 (18.6)	
**Area**			
	South	63 (20.9)	
	East	29 (9.6)	
	West	20 (6.6)	
	North	105 (34.9)	
	Center	38 (12.6)	
	Outside	46 (15.3)	
**Success**			
	Failed	183 (60.8)	
	Passed	118 (39.2)	

^a^n=299. Our respondents completed all variables, except for 2 participants who did not fill the age variable. They were not excluded from the analysis.

There was no significant difference in the percentage of success between the following groups: age group younger than 25 years and 25 years or older; Saudis and non-Saudis; males and females; single, married, and divorced; different degrees of education; college educated or not; different levels of income; living in Jeddah or outside Jeddah; and the area living within Jeddah. There was no significant difference in the exam success rates among any of these groups (all *P*>.05).

**Table 2:** Success rate differences among different variables.

Variable			Success, n (%)			*P* value
			Failed		Passed	
**Age group**						.09
	<25 years	130 (57.8)		95 (42.2)		
	≥25 years	51 (68.9)		23 (31.1)		
**Nationality**						.38
	Saudi	171 (61.5)		107 (38.5)		
	Non-Saudi	12 (52.2)		11 (47.8)		
**Sex**						.19
	Male	56 (66.7)		28 (33.3)		
	Female	127 (58.5)		90 (41.5)		
	Marital status					.41
	Single	142 (59.2)		98 (40.8)		
	Married	40 (66.7)		20 (33.3)		
	Divorced	1 (100.0)		0 (0.0)		
**Educational level**						.21
	Illiterate	1 (50.0)		1 (50.0)		
	High school or less	67 (63.2)		39 (36.8)		
	Professional certificate (noncollege issued)	9 (81.8)		2 (18.2)		
	Bachelor	99 (56.9)		75 (43.1)		
	Masters or PhD	7 (87.5)		1 (12.5)		
**Monthly income (SR)**						.25
	less than 5000	26 (76.5)		8 (23.5)		
	5000 to 10,000	58 (58.0)		42 (42.0)		
	10,000 to 20,000	59 (60.2)		39 (39.8)		
	>20,000	40 (58.0)		29 (42.0)		
**Residence**						.36
	Jeddah	152 (62.0)		93 (38.0)		
	Outside Jeddah	31 (55.4)		25 (44.6)		
**Area**						.85
	South	42 (66.7)		21 (33.3)		
	East	17 (58.6)		12 (41.4)		
	West	12 (60.0)		8 (40.0)		
	North	65 (61.9)		40 (38.1)		
	Center	22 (57.9)		16 (42.1)		
	Outside	25 (54.3)		21 (45.7)		
**College**						.26
	Not college educated	77 (64.7)		42 (35.3)		
	College educated	106 (58.2)		76 (41.8)		

Pearson correlation was done (Table 3) to test for significance between the total score and age and it showed a significant correlation between (*P*=.04). It was a very weak negative correlation (*r*=–.12), meaning the test total score tended to be lower if the participant was of older age. Spearman correlation was done between the total score and the educational level as well as the total score and income level, but there was no significant correlation in either case.

**Table 3:** Correlations between the total score and age, income level, and educational level (N=301).

Total score versus	Correlation coefficient	*P* value
Age^a^	–.119	.04
Income level	.066	.26
Educational level	.052	.37

^a^n=299. Our respondents completed all variables, except for 2 participants who did not fill the age variable. They were not excluded from the analysis.

**Table 4:** Distribution of college-educated participants according to different variables.

Variable		College education, n (%)		*P* value
		Not college educated	College educated	
**Age group**				<.001
	<25 years	103 (45.8)	122 (54.2)	
	≥25 years	15 (20.3)	59 (79.7)	
**Nationality**				.07
	Saudi	114 (41.0)	164 (59.0)	
	Non-Saudi	5 (21.7)	18 (78.3)	
**Sex**				.40
	Male	30 (35.7)	54 (64.3)	
	Female	89 (41.0)	128 (59.0)	
**Marital status**				<.001
	Single	108 (45.0)	132 (55.0)	
	Married	10 (16.7)	50 (83.3)	
	Divorced	1 (100.0)	0 (0.0)	
**Monthly income (SR)**				.07
	<5000	16 (47.1)	18 (52.9)	
	5000 to 10,000	29 (29.0)	71 (71.0)	
	10,000 to 20,000	44 (44.9)	54 (55.1)	
	>20,000	30 (43.5)	39 (56.5)	
**Residence**				.97
	Jeddah	97 (39.6)	148 (60.4)	
	Outside Jeddah	22 (39.3)	34 (60.7)	
**Area**				.40
	South	19 (30.2)	44 (69.8)	
	East	9 (31.0)	20 (69.0)	
	West	8 (40.0)	12 (60.0)	
	North	44 (41.9)	61 (58.1)	
	Center	18 (47.4)	20 (52.6)	
	Outside	21 (45.7)	25 (54.3)	

Being college educated or not was tested among different factors (Table 4). It was only significant with the age group (*P*<.001), and the percentage college educated was found to be higher in the age group older than 25 years. It was also significant for marital status (*P*<.001), where college education was higher in percentage in married participants than in singles. There was no significant difference regarding college education for the other factors, including nationality, gender, income level, residence in Jeddah, and area of living within Jeddah.

**Table 5:** Binary logistic regression result for the score (passed/failed).

Variables		Odds ratio (95% CI)	*P* value
**Nationality**			
	Saudi	0.499 (0.196-1.269)	.14
	Non-Saudi (ref^a^)		
**Sex**			
	Male	0.732 (0.409-1.310)	.29
	Female (ref)		
**Marital status**			
	Single (ref)		.95
	Married	1.163 (0.470-2.877)	.74
	Divorced	0.000 (0.000)	>.99
**Monthly income (SR)**			
	<5000 (ref)		.19
	5000 to 10,000	2.631 (1.033-6.704)	.04
	10,000 to 20,000	2.632 (1.023-6.772)	.05
	>20,000	2.773 (1.045-7.355)	.04
**Residence**			
	Jeddah	0.611 (0.327-1.143)	.12
	Outside Jeddah (ref)		
**Age group**			
	<25 years	1.933 (0.814-4.587)	.14
	≥25 years (ref)		
**College education**			
	College educated	0.724 (0.433-1.210)	.22
	Noncollege educated (ref)		
	Constant	0.554	.42

^a^Ref: reference.

Binary logistic regression was done to test for the effect of various factors on passing the test (Table 5). The Nagelkerke *R*^*2*^for the model was .064, meaning that the model could predict only 6.4% of cases. The only significant odds ratio was in the income groups compared to the group with income less than 5000 SR. The odds ratio for the income between 5000 and 10,000 was 2.63 (95% CI 1.03-6.70), for the group 10,000 SR to 20,000 SR was OR 2.63 (95% CI 1.02-6.77), and for the group with income greater than 20,000 SR, the odds ratio was 2.77 (95% CI 1.04-7.355), meaning that participants with income greater than 5000 SR had a higher chance of passing the exam.

## Discussion

More than two-thirds of the study sample were female (72.1%), which is much higher than the latest census [13]. and even most reported studies [14-16]. The majority of this population (75.3%) were young adults younger than 25 years. The percentage of bachelor’s degrees or higher holders was more than 60%; this may be attributed to the samples being collected in Jeddah, a city with three major universities. Despite this, the sample provided us with insight into a young, educated group, who had more time to gain BLS and CPR knowledge in university as evident by another study done in Riyadh, Saudi Arabia, where the largest number of participants with any CPR knowledge gained it while they were enrolled in university [17].

The number of participants who passed the test was only 118 (39.2%), which is higher than a similar study done in Hong Kong [16], but still less than other studies done in Poland, the United States, and Turkey [18-20]. Even with the higher scores, it does not necessarily mean the ones who passed had any previous BLS training. This can be explained partially by the possibility that some people do have general knowledge without attending BLS courses through courses in school and mainstream media. The American Heart Association has stated that to reduce both morbidity and mortality from OHCA in a significant manner, 20% of adults need to have CPR training [21].

Higher income (more than 5000 SR/month) was associated with higher chance of passing the exam. This can be explained alongside the high education levels: the higher the education level was, the more proportionately higher was the income [22]. However, both income and education were not correlated with higher total score. Age, on the other hand, was negatively correlated with the total BLS score, indicating that younger age was associated with a higher score. Although, it is weakly related, it is nonetheless significant. This may be attributed to forgetting the theoretical part of BLS, especially if more years have passed since they have been exposed to this knowledge. Some hospitals recommend taking refresher courses at least every 3 years [16]. This is due to the diligent aspects of resuscitation and that mistakes in any of its steps may compromise the whole process.

Numerous studies have demonstrated the effects of incorporating BLS support measures by trained regular citizens/laypersons, reducing both the rates of mortality and morbidity [23-25]. Individuals receiving CPR from trained citizens have been found to be four times more likely to live and survive for a month in comparison to those who did not receive CPR [26]. Increasing BLS training centers should be essential to improve the survival rates from OHCA. It should even include schools because it provides an opportunity to expose as much of the population as possible to resuscitation techniques. One region in the United Kingdom introduced its schools to CPR training and it was well received with more than 99% of its students agreeing to its benefit to them [27].

One of the limitations of our study is the small number and scope due to including just one city (Jeddah) in Saudi Arabia, thus the study may not represent the whole population of Jeddah. In comparison to the total Saudi population, our study contained more females and had more participants with a higher education, with generally a younger age group in comparison to the total Saudi population [13]. Another limitation of our study was the inherit bias in how participants decided whether they had a medical background. In addition, it was not possible to determine the response rate of the participants due to using social media sites to distribute the questionnaire. Also, a response was only registered after full completion of the questionnaire, so any person who stopped midway could not be accounted for. We propose more studies to be done that are more comprehensive and include the entire region of Saudi Arabia. Another limitation of this study is that it only tested the participant’s theoretical knowledge; testing it practically was not viable for our study population. Lastly, there was no way to ascertain if any of the population had prior BLS training or if they ever practiced any BLS measures in a real-life setting.

This study demonstrated that the theoretical knowledge level of BLS among the general population in Jeddah was below average. There is a need to increase the public’s exposure to BLS education by raising awareness campaigns and government-funded training programs that aim to curb the incidence of OHCA in the Saudi community.

## Appendix

Multimedia Appendix 1 Questionnaire used in study.

## Abbreviations

ALS: advanced life support
BLS: basic life support
CPR: cardiopulmonary resuscitation
OHCA: out-of-hospital cardiac arrest
OR: odds ratio
