# Supplementary material for: Basic Life Support Knowledge Among a Nonmedical Population in Jeddah, Saudi Arabia: Cross-Sectional Study
Source: Interact J Med Res. 2018 Nov 28;7(2):e10428. doi: 10.2196/10428 (PMC6291680; doi:10.2196/10428)
Supplement: Multimedia Appendix 1 [file ijmr_v7i2e10428_app1.pdf]

The questionnaire below was developed by BLS provider National Health Care Provider Solutions. Permission to use and reprint the questionnaire was taken from them prior to conduction of study.

1. The compression to ventilation ratio for one rescuer giving CPR to individuals of ANY age is:*

- 30:1
- 30:2
- 15:1
- 15:2

2. How often should rescuers switch roles when performing two-rescuer CPR?*

- After every cycle of CPR
- After every two cycles of CPR
- After every five cycles of CPR
- After every 10 cycles of CPR

3. The proper steps for operating an AED are:*

- Power on the AED, attach electrode pads, shock the individual, and analyze the rhythm
- Power on the AED, attach electode pads, analyze the rhythm, clear the individual, and deliver shock
- Attach electrode pads, check pulse, shock individual, and analyze rhythm
- Check pulse, attach electrode pads, analyze rhythm, shock patient

4. Where should you attempt to perform a pulse check in a child who is anywhere from one year to puberty?*

- Brachial artery
- Ulnar artery

- Temporal artery
- Carotid or femoral artery

5. The initial Basic Life Support (BLS) steps for adults are:*

- Assess the individual, give two rescue breaths, defibrillate, and start CPR
- Assess the individual, activate EMS and get AED, check pulse, and start CPR
- Check pulse, give rescue breaths, assess the individual, and defibrillate
- Assess the individual, start CPR, give two rescue breaths, and defibrillate

6. The critical characteristics of high-quality CPR include which of the following?*

- Starting chest compressions within 10 seconds of recognition of cardiac arrest
- Pushing hard and fast
- Minimizing interruptions
- All of the above

7. The five steps in the Adult Chain of Survival include all of the following EXCEPT:*

- Early CPR
- Rapid defibrillation
- Advanced airway placement
- Integrated post-cardiac arrest care

8. The 2015 AHA guidelines for CPR recommended BLS sequence of steps are:*

- Chest compressions, Airway, Breathing
- None of the above

- Airway, Breathing, Check pulse
- Airway, Breathing, Chest compressions

9. Which of the following are signs of airway obstruction?*

- Poor air exchange
- High-pitched noise while inhaling
- Inability to speak
- All of the above

10. In an adult with an advanced airway in place during two-rescuer CPR, how often should the breaths be administered?*

- Every 2 to 3 seconds (20 to 30 breaths per minute)
- Every 4 to 5 seconds (12 to 15 breaths per minute)
- Every 6 to 8 seconds (8 to 10 breaths per minute)
- Every 10 to 12 seconds (5 to 6 breaths per minute)
